# Nutritional support practices and opinions toward gastrostomy use in pediatric bone marrow transplant centers: A national survey

**DOI:** 10.1016/j.nut.2021.111556

**Published:** 2022-03

**Authors:** James Evans, Dan Green, Graeme O Connor, Julie Lanigan, Faith Gibson

**Affiliations:** a Dietetics Department, Great Ormond Street Hospital for Children, London, UK; b University College London Great Ormond Street Institute of Child Health, UK; c Section of Public Health, School of Health and Related Research (ScHARR), University of Sheffield, UK; d School of Health Sciences, University of Surrey, Guildford, UK; e Centre for Outcomes and Experience Research in Children's Health, Illness and Disability (ORCHID), Great Ormond Street Hospital for Children, London UK

**Keywords:** Pediatric, Hematopoietic stem cell transplantation, Gastrostomy, Nutrition support, Enteral nutrition, Survey

## Abstract

*Objective:* Previous surveys have shown deviations in nutritional practices from international guidelines during bone marrow transplant (BMT). Guidelines recommend enteral nutrition first-line and nasogastric tubes are the mainstay for its provision. Gastrostomies provide an alternative, yet their use is less common. This national survey investigated nutrition support practices in pediatric allogeneic BMT centers and compared clinicians’ opinions on gastrostomy use. The aim of this study was to identify the national picture of nutritional support practices across pediatric allogeneic BMT centers, including use and opinions of dietitians, clinical nurse specialists, and physicians, toward gastrostomy feeding.

*Methods:* An online survey was administered to 12 centers. The lead dietitian answered questions regarding nutritional counseling, screening, assessment, and interventions. Questions regarding current use, perceived advantages, and problems of gastrostomies were answered by the dietitian, lead clinical nurse specialist, and physician.

*Results:* A 100% response rate was achieved from 12 centers (N = 36 clinicians). Nutritional counseling was provided in 92% of centers before and routinely throughout admission, 83% screened on and regularly throughout admission, 83% assessed nutritional status before transplant, and 92% used enteral nutrition first-line. Forty-two percent of the centers used gastrostomies. In those not using gastrostomies, 76% of clinicians felt some children should be offered a gastrostomy. Clinicians perceived less displacements (78%) and cosmetic appearance (69%) as the most common advantages of gastrostomies over nasogastric tubes. Risks associated with surgery (92%) and tube/stoma complications (58%) were the most common perceived problems.

*Conclusions:* A similar approach was shown on many aspects of nutritional support. Gastrostomy use divided opinion with differences in use and perceived advantages, but agreement on potential complications. Despite their risks, clinicians wanted to use gastrostomies more. Placement requires careful consideration of the risks, benefits, and family preferences.

## Abstract (author highlights)

• Studies have shown variations in nutrition practices during bone marrow transplant.
• Nasogastric tubes are the mainstay for provision of enteral nutrition.
• Gastrostomy use in this setting is less common.
• Similar approaches were shown for many aspects of nutritional support.
• Despite the risk for infection, they WANTED to increase their use of gastrostomies.

Gastrointestinal (GI) toxicity and mucositis from conditioning regimens, coupled with the risk for graft-vs-host disease (GVHD), put children undergoing allogeneic bone marrow transplant (BMT) at risk for malnutrition [1,2]. Associations have been found between malnutrition and GVHD, survival, transplant-related mortality, and relapse risk [3,4]. Optimal nutritional care is essential for protection from these deleterious outcomes [2].

Nutritional care should be multidisciplinary and consist of counseling, screening, assessment, and monitoring [5,6]. Nutritional support guidelines from the American Society for Parenteral and Enteral Nutrition (ASPEN) and European Society for Clinical Nutrition and Metabolism (ESPEN) [7,8], recommend first-line enteral nutrition (EN) in patients with a functioning GI tract, and parenteral nutrition (PN) reserved for severe mucositis, intractable vomiting, diarrhea, or gut GVHD. Observational pediatric BMT studies have shown first-line ENen, rather than PN, is associated with better day 100 survival, shorter admission [9], less GVHD, and faster platelet engraftment [10].

However, recommendations from ASPEN and ESPEN are based on weak evidence [3,6]. Surveys of nutritional practices [5,6,[11], [12], [13]] have shown deviations from guidelines; absence of standard operating procedures [6]; variations in clinical pathways, decision making [5], and interventions [10], [11], [12]; and many continuing to use first-line PN [9,11].

One similarity across studies is the administration of EN via nasogastric (NG) tubes [14], [15], [16]. Although these can be placed simply, they are susceptible to dislodgement with vomiting and placement refusal [17]. Gastrostomies provide an alternative. They can be more aesthetically acceptable [18], have demonstrated nutritional optimization [19,20] and less use of PN [21], with only minor complications [22], [23], [24]. However, gastrostomy use in BMT remains limited due to the risk for infectious complications [25], despite recommendations that they could be considered given the intensive conditioning and anticipated long-term nutrition support [26].

Previous surveys [5,6] have included only 27% to 37% of centers performing pediatric BMT. No previous survey has explored nutrition support practices in pediatric BMT centers across our country, including current use, barriers, advantages, and disadvantages of gastrostomy feeding in this population.

This study aimed to identify the national picture of nutritional support practices across pediatric allogeneic BMT centers, including use and opinions of dietitians, clinical nurse specialists, and physicians, toward gastrostomy feeding.

## Methods

A survey was developed using the literature [5,6,11,12,[26], [27], [28]], discussions between the research team, and patient and public involvement through focus groups and interviews with children, parents, BMT dietitians, and nurses [25]. Nutritional practices investigated included the following:

• Counseling: who is involved and when is it provided;
• Screening: which clinicians undertake screening; when does it take place; how does it takes place: anthropometry, biochemistry, diet/social history taking, screening tools;
• Assessment and monitoring: current guidelines/protocols; nutritional support teams;
• Interventions: EN and PN; criteria for initiation; barriers to use; and
• Gastrostomies: use, decision making, advantages, and risks.

Content validity was established after review by independent BMT dietitians and physicians. The online survey, designed using SurveyMonkey, was entirely multiple choice (participants were allowed to add free-text comments), and a response was mandatory for all items to avoid missing data. The survey was piloted with dietitians, physicians, and clinical nurse specialists at one center with minor changes made before distribution.

Twelve centers undertaking pediatric allogeneic BMT were identified from the National Society of Blood and Marrow Transplantation [29] and the national pediatric oncology dietitians group and invited to participate. Recruitment and data collection occurred between March and April 2021. Contact details for dietitians are shared across the national group. The lead dietitian within each center was initially e-mailed an explanation of the study, an invitation to participate, and asked to submit contacts for the lead physician and clinical nurse specialist within their center. The physician and clinical nurse specialist were subsequently contacted and invited to participate. If no response was received, a maximum of three follow-up emails were sent. Once participants confirmed consent to participate, the survey link was e-mailed.

One response was required from a dietitian, physician, and clinical nurse specialist within each center. For centers with multiple dietitians, physicians, and clinical nurse specialists, a collective, single response was encouraged from each clinical group to give equal weighting across centers. The dietitian was sent the complete survey containing all nutritional support topics, including opinions about gastrostomies, as the dietitian was felt to be the most appropriate clinician to complete these sections. The physician and clinical nurse specialist were only sent the questions relating to gastrostomy opinions from the dietitians’ survey to allow comparisons between clinicians on this subject. Participants were given 4 wk to complete the survey. A reminder e-mail was sent to non-responders 1 wk before the deadline.

Statistical analyses were performed using SPSS version 27 (IBM, Armonk, NY, USA) with *P* < 0.05 considered statistically significant. Quantitative data consisted entirely of categorical variables expressed as frequencies and percentages. Comparisons between clinician's responses were analyzed using Fisher's exact test due to low expected cell counts.

The study protocol was registered on ClinicalTrials.gov. This research was approved and performed in accordance with the ethical standards of Newcastle and North Tyneside 2 Research Ethics Committee, Integrated Research Application System reference 281830. Informed consent was obtained from all participants.

## Results

### Demographics

A 100% (N = 36) response rate was achieved. No missing data occurred. Nine centers performed 10 to 49 allogeneic transplants annually (75%); 10 transplanted children who had both non-malignant and malignant diseases (83%), and 11 performed matched-related, unrelated, haploidentical, and cord transplants (92%; Table 1).

**Table 1:** Summary demographic data of participating centers (N = 12)

Characteristic	n (%)
Allogeneic transplants/y	
10–49	9 (75)
50–100	3 (25)
Conditions treated	
Malignant and non-malignant, roughly equal numbers	4 (33)
Malignant and non-malignant, roughly more malignant	4 (33)
Malignant and non-malignant, roughly more non-malignant	2 (17)
Only malignant	1 (8)
Only non-malignant	1 (8)
Transplant type performed	
Matched related donors	12 (100)
Matched unrelated donors	12 (100)
Cord blood	12 (100)
Haploidentical donors	11 (92)

### Counseling

Nutritional counseling at preadmission was provided by 11 centers (92%), on admission by 7 (58%), routinely throughout admission by 11 (92%), and after discharge by 6 (50%). Counseling was performed by dietitians in all centers, nurses in 10 centers (83%), and by physicians in 9 centers (75%; Table 2).

**Table 2:** Timing and methods of nutrition counseling, screening, assessment, and monitoring*

Topic (question)	Response options	n (%)
When is nutrition counseling provided?†	Before admission	11 (92)
	On admission	7 (58)
	Routinely throughout admission	11 (92)
	After discharge	6 (50)
Who usually provides nutrition counseling?†	Dietitian	12 (100)
	Nurse	10 (83)
	Physician	9 (75)
When is nutrition screening performed?	Screening on admission and regularly throughout admission	10 (83)
	Screening on admission only	1 (8)
	No screening	1 (8)
In centers where screening takes place, who performs the screening?† (n = 11)	Nurse	9 (82)
	Dietitian	7 (64)
	Physician	3 (27)
In centers where screening takes place, how does this occur?† (n = 11)	Anthropometric parameters	9 (82)
	Part of history taking (social and dietary)	9 (82)
	Specific nutrition tools	7 (64)
	Blood chemistry parameters	4 (36)
When is the nutritional status of children assessed?†	Before transplant	10 (83)
	After discharge, for all children	7 (58)
	After discharge, only for children with nutritional difficulty	3 (25)
	Neither	1 (8)
Does your center have a guideline, protocol, or procedure that specifies how to monitor children's nutritional status?	Yes	5 (42)
	No	7 (58)
In centers with a multidisciplinary nutrition support team, who is part of it?† (n = 9)	Dietitian	9 (100)
	Gastroenterologist	9 (100)
	Parenteral nutrition pharmacist	9 (100)
	Nurse	7 (78)
	Physician (BMT/hematology/oncology)	4 (44)

⁎ N = 12 centers, unless stated otherwise.

† Multiple answers possible. BMT, bone marrow transplant

### Screening

Screening was undertaken by 11 centers (92%), primarily on admission and regularly throughout admission by 10 (83%). Nurses and dietitians most frequently screened patients at 9 (82%) and 7 (64%) centers, respectively. The most popular methods included anthropometry (82%), with weight and weight-related indices including body mass index or percentage weight loss used in all centers, mid-upper arm circumference in one center, and other methods including triceps skinfold thickness and bioelectrical impedance not routinely used in clinical practice; dietary and social history taking (82%), typically through retrospective 24-h diet recall or 3-d averaged intake from nursing food charts or patient-weighed food diaries, coupled with interviews about habitual food intake; and screening tools (64%), with three centers using the Screening Tool for the Assessment of Malnutrition in Pediatrics [30], Screening Tool for the Risk of Impaired Nutritional Status and Growth [31], and Pediatric Yorkhill Malnutrition Score [32] (Table 2). These tools combine scores from three to four questions including the nutritional effect of the child's diagnosis or clinical status, assessment of nutritional intake, current weight and height, and weight trends over the preceding weeks or months. The combined score from these questions places the child at either high risk, where dietetic referral is recommended; medium risk, where monitoring of intake and repetition of screening is advised after 3 d; or low risk where current care continues, and screening is repeated weekly.

### Assessment, monitoring, and nutritional support teams

Children received nutritional assessment pretransplant in 10 of the 12 centers (83%) centers and after discharge (for all children) in 7 (58%). Seven of the 12 centers (58%) did not have a guideline, protocol, or procedure specifying how to monitor children's nutritional status. Nine (75%) had a multidisciplinary nutritional support team. In two centers this was a hospital-wide team only reviewing BMT patients on request. Although every center had a specialist cancer/BMT dietitian, in the 9 centers with a nutritional support team, all included a dietitian (not necessarily the cancer/BMT specialist), PN pharmacist, and gastroenterologist; 7 included a clinical nurse specialist (78%), and 4 a cancer/BMT physician (44%; Table 2).

### Interventions

Eleven of the 12 centers used EN as first-line intervention (92%), with 5 centers (42%) using whole protein, 5 (42%) using hydrolyzed protein, and 2 (17%) using amino acid formulas first-line. EN products used depended on individual center contracts. Whole protein formulas used for children <1 y of age included first infant formulas such as Aptamil 1/Danone: 0.66 kcal/1 mL, 1.3 g protein/100 mL or high-energy formulas including Similac High Energy/Abbott: 1 kcal/1 mL, 2.6 g protein/100 mL, and children >1 y of age Pediasure/Abbott: 1 kcal/1 mL, 2.8 g protein/100 mL, Frebini/Fresenius, 1 kcal/1 mL, 2.5 g protein/100 mL, or Fortini/Nutricia: 1.5 kcal/mL, 3.4 g protein/100 mL. Hydrolyzed protein formulas used for children <1 y of age included Aptamil Pepti-Junior/Nutricia, 0.66 kcal/1 mL, 1.8 g protein/100 mL or Infatrini Peptisorb/Nutricia: 1 kcal/1 mL, 2.6 g protein/100 mL, and children >1 y Pediasure Peptide/Abbott: 1 kcal/1 mL, 3 g protein/100 mL or Nutrini Peptisorb/Nutricia: 1 kcal/mL, 2.8 g protein/100 mL. Amino acid formulas used for children <1 y of age included Neocate LCP/Nutricia: 0.67 kcal/1 mL, 1.8 g protein/100 mL or Puramino/Mead Johnson: 0.68 kcal/1 mL, 1.9 g protein/100 mL, and children from 1 to 18 y of age Neocate Junior/Nutricia: 1 kcal/1 mL, 2.8 g protein/100 mL or Elemental 028 Extra/Nutricia: 0.89 kcal/1 mL, 2.5 g protein/100 mL. Ten of the 12 centers (83%) initiated EN when children met <50% of oral nutritional requirements and 9 of them (75%) when children lost 5% to 10% of their weight from admission. Criteria for initiating PN included intractable vomiting/diarrhea with EN, gut GVHD and inability to advance EN due to tolerance (each present in all 12 of the centers), and meeting <50% requirements from oral/EN (n = 9 [75%]; Table 3). Two of the 12 centers (17%) used prophylactic PN in children with severe faltering growth pretransplant, gastroenteropathy, and cord transplants.

**Table 3:** Interventions used to provide nutritional support and indications for use (N = 12 centers)

Topic (question) and possible responses	n (%)
Which intervention is used to provide first-line nutrition support?	
Enteral nutrition	11 (92)
Parenteral nutrition	1 (8)
What products are used to provide first-line enteral tube feeding?	
Whole protein feeds	5 (42)
Hydrolyzed (partially or extensively) protein feeds	5 (42)
Amino acid feeds	2 (17)
What indications would lead to the initiation of enteral tube feeding?*	
Consume <50% nutritional requirements orally	10 (83)
Weight loss 5%–10% from admission	9 (75)
Weight loss >10% from admission	8 (67)
Consume 50%–75% nutritional requirements orally	6 (50)
What indications would lead to the initiation of parenteral nutrition?*	
Intractable vomiting/diarrhea	12 (100)
Gut graft-versus-host disease	12 (100)
Inability to advance enteral feeds due to tolerance issues	12 (100)
Meeting <50% nutritional requirements from oral and/or tube feeding	9 (75)
Mucositis, grade 3–4	8 (67)
Meeting 50%–75% nutritional requirements from oral and/or tube feeding	3 (25)
Weight loss >10% from admission	2 (17)
Weight loss 5%–10% from admission	1 (8)
Does your center offer children a prophylactic gastrostomy?	
All children are offered a gastrostomy	0 (0)
Some children are offered a gastrostomy	5 (42)
No children are offered a gastrostomy	7 (58)
In what circumstances are prophylactic gastrostomies placed?* (n = 5 centers)	
Poor nutritional status before transplant	5 (100)
Likely to refuse nasogastric tube during transplant	4 (80)
Total body irradiation/myeloablative conditioning	4 (80)
Specific conditions (e.g., Hurler syndrome, severe autism with feeding difficulties)	2 (40)

⁎ Multiple answers possible.

### Barriers to enteral nutrition

Dietitians, clinical nurse specialists, and physicians reported the same most common barriers; NG tube dislodgement (89%), diarrhea/vomiting with tube feeds (83%), and NG tube refusal (78%). Half of the clinical nurse specialists, but none of the dietitians, reported mechanical tube complications as a barrier (*P* = 0.014; Table 4).

**Table 4:** Barriers routinely faced with enteral tube feeding

Barrier	Total n (%)(N = 36)	Dietitian, n (%) (n = 12)	Nurse n (%) (n = 12)	Physician n (%) (n = 12)	*P-*value
Dislodgement with vomiting or pulled out	32 (89)	10 (83)	12 (100)	10 (83)	0.516
Vomiting/Diarrhea during tube feeds	30 (83)	10 (83)	12 (100)	8 (67)	0.131
Refusal of NG tube placement	28 (78)	10 (83)	10 (83)	8 (67)	0.683
Placement contraindication during mucositis and/or thrombocytopenia	19 (53)	7 (58)	7 (58)	5 (42)	0.764
Child in discomfort when NG tube in situ	17 (47)	4 (33)	8 (67)	5 (42)	0.338
Perceived poor tolerance to EN	17 (47)	6 (50)	6 (50)	5 (42)	>0.999
Mechanical tube complications	9 (25)	0 (0)	6 (50)	3 (25)	**0.014**
Epistaxis with NG tubes	7 (19)	3 (25)	3 (25)	1 (8)	0.656
Perceived preference for PN between other multidisciplinary team members	6 (17)	2 (17)	4 (33)	0 (0)	0.131
Differences of opinion regarding tube feeding within the multidisciplinary team	5 (14)	1 (8)	4 (33)	0 (0)	0.101

EN, enternal nutrition; NG, nasogastric; PN, parenteral nutrition

*P* ≤ 0.05 are marked in **bold**

### Gastrostomies

The choice of a prophylactic gastrostomy, placed before transplant, was offered to some children (typically <5–15% of children transplanted annually) in 5 of the 12 centers (42%), most commonly those with poor nutritional status pretransplant (100%), and likely to refuse nasogastric tubes (80%; Table 3). The BMT physician, clinical nurse specialist, parents, and children were involved in the decision to place a gastrostomy in all 5 centers. The dietitian was involved in 4 of the 5 centers (80%), a gastroenterologist/surgeon in 3 centers (60%), a gastrostomy clinical nurse specialist in 2 (40%), and a psychologist, play specialist, and referring physician each in 1 (20%).

Opinions and concerns regarding gastrostomy use across 7 of the 12 centers (58%) not using them are shown in Table 5. The main reasons given by 21 clinicians that gastrostomies were not used in these centers included a tradition of using NG tubes by 18 (86%); risk for complications by 12 (57%); and feeling that gastrostomies were not necessary as current interventions were successful by 11 (52%). Despite these concerns, only 2 (10%) felt that no child should be offered a gastrostomy; however, 16 (76%), including 6 of the 7 dietitians (86%), 5 of the 7 physicians (71%), and 5 of the 7 clinical nurse specialists (71%) felt some children, whereas 3 of the 21 (14%) felt all children should be offered a gastrostomy. Of the 19 who felt some or all children should be offered a gastrostomy, all felt a BMT physician and parents should be involved in the decision, 18 (95%) felt a dietitian and the child should be involved, and 17 (90%) said a BMT clinical nurse specialist should be included in the decision making.

**Table 5:** Opinions and concerns of clinicians (N = 21) regarding gastrostomy use in centers (n = 7) not using this intervention

Topic (question) and possible responses	n (%) (N = 21)
Why are children not offered the choice of a prophylactic gastrostomy?*	
Traditionally use NG tubes	18 (86)
Risk for complications (e.g., infections)	12 (57)
Not necessary; our current methods are successful	11 (52)
Surgery is an additional burden	8 (38)
Never considered this an option	2 (10)
Expertise/Infrastructure not available	1 (5)
Do you think children should be offered a prophylactic gastrostomy?	
All children should be	3 (14)
Some children should be in certain circumstances	16 (76)
No children should be	2 (10)
Who should be involved in the decision to place a gastrostomy?*	*n* (%) (*n* = 19)
Physician (BMT/hematology/oncology)	19 (100)
Parent	19 (100)
Child	18 (95)
Dietitian	18 (95)
BMT/hematology/oncology clinical nurse specialist	17 (90)
Gastroenterologist/surgeon	10 (53)
Gastrostomy clinical nurse specialist	9 (47)
Psychologist	9 (47)
Play specialist	8 (42)
Speech and language therapist	2 (11)

⁎ Multiple answers possible. BMT, bone marrow transplant; NG, nasogastric

Clinicians across all centers perceived less tube displacements (78%), better cosmetic appearance (69%), and ease/safety of providing nutrition/medicines/fluids (64%) as the most common advantages of gastrostomies over NG tubes (Table 6). All of the dietitians and only 5 of the 12 physicians (42%) felt gastrostomies provided a cosmetic advantage (*P* = 0.005) and greater ease/safety of providing nutrition (n = 10 [83%] versus n = 4 [33%], respectively; *P* = 0.036). Seven of 12 clinical nurse specialists (58%) compared with only 1 dietitian felt less tube blockages (*P* = 0.027) was an advantage. Nine clinical nurse specialists (75%) compared with only 3 physicians (25%) felt overnight feeding (*P* = 0.039) was an advantage. Overnight feeding was also perceived as more advantageous in 12 of 15 centers offering gastrostomies (80%) compared with 7 of the 21 not offering the procedure (33%; *P* = 0.008). Clinicians felt similarly about perceived gastrostomy problems. The most common included risk for surgery (92%), tube/stoma complications (58%), and the additional burden on families to care for the gastrostomy (58%). Six of the 21 centers (29%) not offering gastrostomies felt their use posed a greater cost to health services whereas none of the 15 centers using them had the same concerns (*P* = 0.030).

**Table 6:** Perceived advantages and problems of gastrostomies compared with nasogastric tubes

	Total n (%) (N = 36)	Dietitian n (%) (n = 12)	Nurse n (%) (n = 12)	Consultant n (%) (n = 12)	*P*-value	Clinicians in centers offering a gastrostomy, n (%) (n = 15)	Clinicians in centers not offering a gastrostomy, n (%) (n = 21)	*P*
Advantages								
Less tube displacements/reinsertions	28 (78)	11 (92)	10 (83)	7 (58)	0.210	14 (93)	14 (67)	0.104
Better cosmetic appearance	25 (69)	12 (100)	8 (67)	5 (42)	**0.005**	10 (67)	15 (71)	>0.999
Long-term provision of nutrition/medicines/fluids	25 (69)	9 (75)	10 (83)	6 (50)	0.281	12 (80)	13 (62)	0.295
Ease/safety of providing nutrition/medicines/fluids	23 (64)	10 (83)	9 (75)	4 (33)	**0.036**	12 (80)	11 (52)	0.159
More comfort/convenience	20 (56)	7 (58)	7 (58)	6 (50)	>0.999	8 (53)	12 (57)	>0.999
Less risk for aspiration	20 (56)	6 (50)	7 (58)	7 (58)	>0.999	10 (67)	10 (48)	0.320
Less interference in daily activities	20 (56)	5 (42)	7 (58)	8 (67)	0.589	8 (53)	12 (57)	>0.999
Option of overnight feeding at home	19 (53)	7 (58)	9 (75)	3 (25)	**0.039**	12 (80)	7 (33)	**0.008**
Better quality of life	16 (44)	6 (50)	6 (50)	4 (33)	0.762	8 (53)	8 (38)	0.500
Less blockages due to shorter length	12 (33)	1 (8)	7 (58)	4 (33)	**0.027**	6 (40)	6 (29)	0.499
Shorter feeding times	4 (11)	0 (0)	4 (33)	0 (0)	0.093	2 (13)	2 (10)	>0.999
Cost saving to National Health Service	3 (8)	1 (8)	2 (17)	0 (0)	0.758	2 (13)	1 (5)	0.559
Problems								
Risk for surgery for placement	33 (92)	12 (100)	9 (75)	12 (100)	0.092	14 (93)	19 (91)	>0.999
Risk for complications (e.g., infection)	21 (58)	6 (50)	7 (58)	8 (67)	0.911	10 (67)	11 (52)	0.501
Burden on family to care for gastrostomy	21 (58)	5 (42)	7 (58)	9 (75)	0.315	6 (40)	15 (71)	0.090
Negative effect on body image	11 (31)	2 (17)	4 (33)	5 (42)	0.539	4 (27)	7 (33)	0.729
It won't be used/needed	10 (28)	4 (33)	4 (33)	2 (17)	0.717	3 (20)	7 (33)	0.468
Greater cost to National Health Service	6 (17)	3 (25)	1 (8)	2 (17)	0.852	0 (0)	6 (29)	**0.030**
Less comfort/convenience	5 (14)	1 (8)	1 (8)	3 (25)	0.584	2 (13)	3 (14)	>0.999
More interference in daily activities	1 (3)	0 (0)	0 (0)	1 (8)	>0.999	1 (7)	0 (0)	0.417
Worse quality of life	1 (3)	0 (0)	0 (0)	1 (8)	>0.999	0 (0)	1 (5)	>0.999

*P* ≤ 0.05 are marked in **bold**

## Discussion

### Nutritional counseling, screening, and assessment

Malnutrition during BMT is negatively related to patient survival, transplant-related mortality, and relapse compared with those who maintain a good nutritional status throughout transplant [3]. Families should receive nutrition counseling, screening, and assessment early and regularly throughout transplant to stress the importance of nutrition, detect nutritional deficiencies, and prepare them for interventions [7,8,26]. Contrary to other studies, this survey found a proactive, rather than reactive, approach to these issues. Most centers counseled (92%) and screened (83%) on/or before and routinely throughout admission and undertook nutritional assessment pretransplant (83%) and routinely after discharge (58%). Other studies found counseling to be poorly structured, occurring at random, and implemented only as required, with 23% to 57% counseling routinely during admission [5,6]. Variability in screening and assessment has also been shown with 36% to 100% of centers screening [5,6,11] and 57% assessing nutritional status pretransplant [6] and 21% to 49% after discharge [5,6].

Counseling, screening, and assessment should be performed by trained staff to ensure consistent and credible advice [26,33]. Dietitians, physicians, and nurses counseled in 75% to 100% of centers. Specialist professionals, such as dietitians, have been heavily involved in other studies [11,26,27]. Screening was predominantly undertaken by nurses (82%) and dietitians (64%), whereas physicians predominantly screened in 65% of European Society for Blood and Marrow Transplantation (EBMT) centers, as did 35% of dietitians and 22% of nurses [6].

Similar parameters were used to assess nutritional status, most commonly anthropometry and diet/social history taking; similar strategies to other studies [5,6,27,28]. Biochemistry was used less often (36%) than other surveys; 63% to 87% [6,28,34]. Many centers (64%) used screening tools: >16% across EBMT and other centers [5,6,26]. To our knowledge, there is no standardized method for assessing nutritional status in children with cancer and those undergoing BMT [6,[35], [36], [37]]. Consequently, inconsistent strategies are widely used, each having limitations. Anthropometry and albumin are confounded by fluid status and inflammation [36,38], and the Nutrition Screening Tool for Childhood Cancer [39] is the only validated tool in pediatric cancer. Only 42% of centers had a protocol for monitoring patient's nutritional status, similar to other surveys (43% to 56%) [5,6], yet 75% had a multidisciplinary nutritional support team, which was higher than EBMT (35%) and ASIA Pacific Blood and Marrow Transplantation centers (53%) [6,28]. Dietitians, PN pharmacists, gastroenterologists, and nurses featured prominently in teams. This range of clinicians seemed consistent across studies [5,6], although pharmacists were less prevalent among ASIA Pacific Blood and Marrow Transplantation teams (38%) [28].

### Nutritional interventions

Most centers (92%) used EN first-line, rather than PN and initiated it proactively under similar criteria; >5% weight loss and/or intake providing <75% requirements. However, many centers initiated EN before these criteria to familiarize the child with tube feeding and promote acceptability. Indeed, NG tube refusal was a common barrier. Studies have reported children's perceptions of NG tubes as invasive and painful [40], [41], [42]. Integrating systematic placement into protocols on day +1 post-BMT avoids contraindication by mucositis, aids tolerance, and reduces discrepancy in practice between centers [26]. The success of proactive placement depends on a committed multidisciplinary team [15] and preadmission counseling to facilitate NG tube acceptance [12,43]. This approach has shown 50% to 95% of units reporting NG tube tolerance [12].

Implementation of an appropriate and patient-centered enteral feeding regimen will also promote acceptability and success of EN. On reduction or cessation of oral intake during admission, daytime bolus feeds can be introduced to replace or top-up meals, given via gravity or pump over 30 to 60 min, as needed, to promote tolerance. Such regimens can be continued once tolerance is established, or boluses extended to continuous 12- to 20-h pump feeds if vomiting and/or diarrhea are problematic. After engraftment and amelioration of mucositis, continuous pump feeds can gradually be transitioned to daytime boluses, given after or between meals, in preparation for discharge. Once home, children fed via NG tube will have to remain, if needed, on a daytime bolus feeding regimen until their oral intake is sufficient to stop tube feeding. This is because in our country, community NG tube feeding policy largely prohibits overnight feeding due to the risk for tube dislodgement and feed aspiration. However, use of overnight gastrostomy feeding is permitted and often a popular regimen with families [21]. This allows children to consume what they can manage orally during the day with freedom from tube feeds and be topped up overnight with a 9- to 12-h continuous pump feed, as needed. Interestingly this benefit was perceived as more advantageous in centers offering gastrostomies than in those not.

Variation in EN products existed with whole protein and hydrolyzed feeds each used first-line in 42% of centers. Whole protein seemed to be used predominantly in pediatric BMT studies [9,15,21,[43], [44], [45]], with others moving onto hydrolyzed or amino acid products during GI toxicity [21,43]. Absence of trials comparing products could explain variations in practice. Use of first-line hydrolyzed or amino acid feeds, over whole protein, may be advantageous after conditioning, mucositis, and suboptimal nutrient absorption, in ameliorating vomiting/diarrhea and assisting EN tolerance [46]; a common barrier faced by clinicians. Other barriers included NG tube dislodgement and perceived poor tolerance to EN; as seen in other studies [12,28]. One study perceived preference for PN between the multidisciplinary team as a less prominent barrier [12], whereas another study demonstrated that dietitian availability was a barrier that was less prominent [27]. With multidisciplinary teamwork apparently central to most centers surveyed, and with all including a dietitian, it appears there was a collaborative approach leading to fewer differences of opinion.

This proactive approach to EN seems contradictory to other surveys where PN was predominantly used first-line in 50% to 70% of institutions [5,11,12]. Centers surveyed reserved PN for similar reasons including severe mucositis, intake providing <50% requirements, inability to advance EN due to tolerance, gut GVHD, and intractable vomiting/diarrhea; criteria broadly similar to ASPEN and ESPEN guidelines [7,8]. Only 17% of centers used prophylactic PN. Such use varied from 0% [12], to 22% [47], 25% across EBMT [6], and 100% across ASIA Pacific Blood and Marrow Transplantation centers [28]. Traditionally, PN has been the intervention of choice [48]. However, this paradigm has shifted toward EN, which is now established as feasible in pediatric BMT [14,43,49], and associated with better survival, shorter admission [9], reduced incidence of bloodstream infections [50], faster platelet engraftment, and less GVHD [10], than first-line and exclusive PN. Indeed, a recent systematic review also showed EN, rather than PN, reduced the incidence of grade III to IV and gut acute GVHD [1]. Given emerging evidence regarding the association between alterations in the gut microbiome and acute GVHD onset, this protective effect could be attributable to the improved gut microbiota observed post-BMT in patients enterally fed [51,52], and should further encourage the use of first-line EN.

### Gastrostomies

Despite recommendations that gastrostomies could be considered in children undergoing intensive treatment [26], only 42% of centers offered some children (primarily those with preexisting malnutrition or likely to refuse NG tubes), a prophylactic gastrostomy, yet 76% of clinicians in centers not offering gastrostomies felt some children should be offered one. No demographic differences existed between centers offering, and not offering, gastrostomies. All centers agreed decision making should be multidisciplinary, involving BMT physicians, clinical nurse specialists, parents, children, dietitians, and gastroenterologists, the latter especially important to ensure proper gastrostomy management, which could be important in the overall survival rate. It is important that children, whenever possible, are involved as they want decisions regarding nutrition support to be their choice [40]. Centers offering, and not offering, gastrostomies felt similarly about their advantages. One difference, however, was overnight feeding, which community policy generally prohibits via NG tube, but not gastrostomy, as previously discussed. Clinicians disagreed on advantages of less tube blockages and ease/safety of providing nutrition, which likely represent the involvement of dietitians/clinical nurse specialists more than physicians on these issues. The cosmetic benefit of gastrostomies over NG tubes was recognized most by dietitians and has been acknowledged elsewhere [18,22]. Clinicians agreed on similar concerns, most notably the risk for complications, which was a prominent reason for centers not using gastrostomies. However, the evidence for this is mixed. Although one study found significantly more gastrostomy infections in children undergoing BMT [53], others have shown minor complications [20,21,24] and improvement/stabilization of nutritional status [19,21,23,[54], [55], [56], [57]]. Interestingly, more clinicians from centers not offering, compared with those offering gastrostomies, felt they posed an additional burden. Hearing families’ perspectives would elucidate this.

### Limitations

This study included some limitations such as results being based on a survey rather than on clinical observation. There may be differences between clinical practice and what is reported using this method. Only one dietitian, clinical nurse specialist, and physician completed the survey and responses may have reflected individual opinions. Differing views may exist between members of the same profession. However, participants were encouraged to reflect on a shared perspective of practice. Every center surveyed also had a dietitian involved in the management of these children and results could be biased toward proactive nutritional practices. Not all centers will have dedicated dietitians and may be less attentive to nutritional management. Finally, the survey was bespoke, not validated, and did not investigate the full scope of nutritional practices.

## Conclusion

Pediatric BMT centers employ proactive and similar approaches to nutritional counseling, screening, assessment, and interventions. Gastrostomy use divided opinion with differences in use and perceived advantages, but agreement on potential problems. It remains a vexed issue, yet an intervention that clinicians reported to want to use more. Placement requires careful consideration of the risks, benefits, and family preferences. The coordinated work of the multidisciplinary BMT and clinical nutrition teams may be an important factor that increases the chance of gastrostomy acceptance. Future research should focus on how best to evaluate children's nutritional status. Centers should incorporate strategies to overcome barriers to EN and improve its acceptability. Consideration of gastrostomy use in children likely to refuse NG tubes could help this. We are currently recruiting to a mixed methods study investigating complications, outcomes, and family experiences of gastrostomy feeding in pediatric BMT. We hope this will persuade centers apprehensive of using gastrostomies to consider them as a preferential alternative to NG tubes for certain children.
